# The Thirty-First Annual Commencement of the Baltimore College of Dental Surgery

**Published:** 1871-03

**Authors:** 


### EDITORIAL DEPARTMENT.

---

*The Thirty-first Annual Commencement of the Baltimore College of Dental Surgery*, was held Thursday evening, March 2d, 1871, in the Concordia Opera House. The spacious Hall was crowded with as brilliant an audience as ever assembled on a similar occasion, many being obliged to stand; and altogether the scene was of that attractive nature which scarcely any occasion except a college commencement could have produced. Holland's Blues Band entertained the large assembly with a number of appropriate musical selections. Upon the stage were seated the members of the Faculty, the graduating class, and a large number of guests, among them the Deans and several Professors of the two medical colleges of this city; while the front of the stage was strewed with boquets, intended as offerings of esteem and friendship from the many lady friends of the graduates.

The exercises were opened with prayer by the Rev. E. R. Eschbach, D. D., after which Prof. F. J. S. Gorgas, M. D., Dean of the Faculty, read the authority for conferring the degree of Doctor of Dental Surgery, from the Charter of the Institution, granted in 1839, by the Legislature of the State of Maryland, and announced the names of the graduates.

Professor Gorgas then conferred the degree of "Doctor of Dental Surgery" upon the following gentlemen:

Edward William Anderson........................ *Virginia.*
Mintern S. Brown.................... *District of Columbia.*
Oliver A Dailey, M. D................*District of Columbia.*
John Hiram Darrell..................*District of Columbia.*
Charles William Fisher.................................. *Texas.*
William J. Fogle..........................................*Georgia.*
Frank Lewis Harris.................................. *Virginia.*
James Richard Harvey.............................*Arkansas.*

Thomas Milton Howard........................................*Georgia.*
Exum Lewis Hunter..............................*North Carolina.*
Robert Green Hunter.........................................*Florida.*
Henry Cabell Jones........ ............................. *Virginia*
Robert Edward King............................*North Carolina.*
Thomas Berwick Legare.......................*South Carolina.*
Francis Hilleary Mannakee..........................*Maryland.*
William A. Mills............................................. *Virginia.*
John Marshall Norman................................ *Tennessee.*
Ambrose Schuyler Page.............................. *Virginia.*
Thomas Henson Parramore......................... *Virginia.*
John Francis Poulton.................................. *Virginia.*
Joseph Henry Scales.................................... *Virginia.*
Samuel Isaac Scott......................................*Maryland.*
James Hosea Sherwood..............................*Maryland.*
Edward Hopkinson Stelle...........*District of Columbia.*
William Luois Stewart..........................................*Texas.*
Lawson Browning Wilson...........................*Maryland.*
George S. Yingling, M. D......................................*Ohio.*

After the regular degrees were conferred, Professor P. H. Austen announced that although the *Gradus Honorarius* of Doctor of Dental Surgery had not been conferred since 1860, the Faculty would deviate from the rule by conferring this degree upon Norman William Kingsley, of New York, for "Scientific Investigation of Deficiencies of the Palate, and the application of a very remarkable artistic skill to the Artificial Replacement of the same."

This degree, which, after an interval of ten years, the College has resumed the right occasionally to confer, must necessarily be unsolicited, and will always be given in recognition of special service, and not of general excellence or high standing.

The College will under no circumstances take cognizance of, or feel called upon to reply to an *application* for its Honorary Diploma.

The following is a copy of the Diploma conferred on Dr. Kingsley:

THE BALTIMORE COLLEGE OF DENTAL SURGERY.

To all whom it may concern.

*Whereas:* It is a chartered right and privilege of this Institution to confer the seal of approbation upon any important service rendered to the Science and Art of Dentistry; or upon any addition to its means of relieving human suffering, and

*Whereas:* NORMAN WILLIAM KINGSLEY, of the City and State of New York, and United States of America, by a Scientific investigation of *Congenital Deficiencies* of the *Palate*, and by the application of a very remarkable artistic skill to the *Artificial*

*Replacement* of the same, has demonstrated the high capabilities of Dental Art and has rendered invaluable service to this unfortunate class ot sufferers:

*Now therefore,* We, the *Faculty of the Baltimore College of Dental Surgery,* in acknowledgement of these services rendered to Dentistry and to Humanity, do hereby confer upon the said NORMAN WILLIAM KINGSLEY, the *Gradus Honorarius* of our Institution with all the privileges and immunities thereunto belonging.

In testimony whereof, we have caused to be executed this DIPLOMA of MERIT and have thereunto severally set our hands and the Corporate Seal of our College, on this second day of March, in the year of our Lord, one thousand eight hundred and seventy one.

| | |
|---|---|
| Given at the City of<br>Baltimore, State of Maryland,<br>United States of America. | Signed by the Faculty,<br>with<br>Seal of College attached. |

The Valedictory Address was then delivered by Professor Thomas E. Bond, M. D., who humorously referred to the manner in which each professor was expected to take his turn in the delivery of the valedictory, took up the subject of man, and spoke of him as a "moral and intellectual animal. The error of life, he said, was that we were apt to look at and deal with ourselves in sections. We were apt to talk of the soul, body and spirit as being separate and divided. Not so, he said, they were a unit. The great difficulty was in not seeing it in this light.

People were too apt to look upon Sunday as the soul's day—hence some persons only practice religion on that day. As the graduates were going out into the world to assume the responsibilities of men, it was important, he said, to start right. This was a fast age, God knows it is, and so does the devil. The trouble was that the whole community was drifting too much into animalism, were living too much apart from the intellectual and moral portion of their being. This, he said, was found as much among the ladies as among men. Women were given too much to decorating themselves with porcupine quills and red tape.

In starting out into the world they should remember that they had each a soul, and the great attribute of the soul was love. They should remember all the time that they had a soul during the week as well as on Sunday, and even a dentist should do this, for though a dentist may have no feeling, he has a soul. They should remember that they had something that the monkey has not. As the soul had but one attribute, love, they should soon turn their love towards God.

The doctor concluded by exhorting the graduates to begin right in this world, so as to end in imperishable glory in a brighter world. He was attentively listened to by the large audience throughout.

Following the remarks of Professor Bond, an appropriate address was delivered in an able manner, by Henry Cabell Jones, of the graduating class, which we publish in the present number of the *Journal.* The exercises, which were highly interesting throughout, were closed with the benediction by the Rev. Dr. Eschbach.

The next session of the College will commence on the 16th of October, 1871.

